# Supplementary material for: Efficacy and safety of eszopiclone combined with drug therapy in the treatment of insomnia after stroke: A network meta-analysis and systematic review
Source: PLoS One. 2024 Feb 5;19(2):e0297064. doi: 10.1371/journal.pone.0297064 (PMC10843102; doi:10.1371/journal.pone.0297064)
Supplement: S2 Appendix — (DOC) [file pone.0297064.s003.doc]

binary data

Set

network setup d n, studyvar(study) trtvar(trt) ref(A) or

network convert pairs

networkplot _t1 _t2, labels(A B C D E )

global consistency

network convert augment

network meta inconsistency

network convert pairs

ifplot _y _stderr _t1 _t2 study,eform labels(A B C D E )

network convert augment

network sidesplit all

network convert augment

network meta consistency

Network prediction interval graph

intervalplot, eform null (1) labels (A B C D E)

League Chart:

netleague, eform labels (A B C D E)

SUCRA

network rank max, all zero seed(10001) reps(10001) meanrank gen(prob)

sucra prob*,labels(A B C D E)

Publication bias

network convert pairs

netfunnel _y _stderr _t2 _t1, random bycomparison

Continuous variable

network setup mean sd n, studyvar(study) trtvar(trt) md ref(1)

network convert pairs

networkplot _t1 _t2, edgesc(1.2) asp(0.8) labels(A B C D E)

global consistency

network convert augment

network meta inconsistency

Local inconsistency

network convert pairs

ifplot _y _stderr _t1 _t2 study, labels(A B C D E F G)

network convert augment

network sidesplit all

network convert augment

network meta consistency

Network prediction interval graph

intervalplot, null (0) labels (A B C D E) separate margin (10 20 5 10) textsize (1) xlabel(-3 -1 0 1 3)

SUCRA

network rank min, seed (10001) reps(10001) meanrank gen(prob)

sucra prob*,labels(A B C D E)

network convert pairs

netfunnel _y _stderr _t2 _t1, random by comparison

League Chart:

network convert augment

netleague, labels (A B C D E )
