# Supplementary material for: Efficacy and safety of eszopiclone combined with drug therapy in the treatment of insomnia after stroke: A network meta-analysis and systematic review
Source: PLoS One. 2024 Feb 5;19(2):e0297064. doi: 10.1371/journal.pone.0297064 (PMC10843102; doi:10.1371/journal.pone.0297064)
Supplement: S1 Table — (DOC) [file pone.0297064.s004.doc]

| Author | Patient nationality | disease duration(M: month; D:day) | | | | | | |
| --- | --- | --- | --- | --- | --- | --- | --- | --- |
|  |  | I | | | | C | | |
| Cheng GH [11] | China | 6. 20 ± 1. 32M | | | | 6. 31 ± 1. 35M | | |
| Guo YZ [12] | China | - | | | | - | | |
| He M [13] | China | - | | | | - | | |
| Hou CY [14] | China | 21.7 3.7D | | | | 21.2 3.9D | | |
| Huang WZ [15] | China | - | | | | - | | |
| Li HS [16] | China | - | | | | - | | |
| Lu F [17] | China | 6.57 3.48M | | | | | | |
| Lv X [18] | China | - | | | | - | | |
| Song YM [19] | China | - | | | | - | | |
| Wang SH [20] | China | - | | | | - | | |
| Wang Y [21] | China | Duration | 1-6M | 6-12M | >12M | 1-6M | 6-12M | >12M |
|  |  | number | 12 | 15 | 18 | 12 | 16 | 19 |
| Wang YF [22] | China | - | | | | - | | |
| Wei QZ [23] | China | 8.13 1.2D | | | | 8.21 1.17D | | |
| Xiao DF [24] | China | - | | | | - | | |
| Yang M [25] | China | 8.05 2.11D | | | | 8.11 1.83D | | |
| Zhang KM [26] | China | - | | | | - | | |
| Zheng YH [27] | China | 2.25 0.31M | | | | 2.31 0.33M | | |
| Zhu ZQ [28] | China | 4.6 0.58M | | | | 4.61 0.57M | | |
