# Supplementary material for: Efficacy and safety of eszopiclone combined with drug therapy in the treatment of insomnia after stroke: A network meta-analysis and systematic review
Source: PLoS One. 2024 Feb 5;19(2):e0297064. doi: 10.1371/journal.pone.0297064 (PMC10843102; doi:10.1371/journal.pone.0297064)
Supplement: Attachment — Submitted filename: rebuttal letter.docx [file pone.0297064.s006.docx]

Dear editor and reviewers

1. Please ensure that your manuscript meets PLOS ONE's style requirements, including those for file naming.

**Reply: I have adjusted the format of the document according to the requirements.**

2. Note from Emily Chenette, Editor in Chief of PLOS ONE, and Iain Hrynaszkiewicz, Director of Open Research Solutions at PLOS: Did you know that depositing data in a repository is associated with up to a 25% citation advantage (https://doi.org/10.1371/journal.pone.0230416)? If you’ve not already done so, consider depositing your raw data in a repository to ensure your work is read, appreciated and cited by the largest possible audience. You’ll also earn an Accessible Data icon on your published paper if you deposit your data in any participating repository

**Reply: I have uploaded the data as a supporting information file.**

3. Please include captions for your Supporting Information files at the end of your manuscript, and update any in-text citations to match accordingly. Please see our Supporting Information guidelines for more information:

**Reply: I have referenced the supporting information file according to your request.**

4. Kindly register your study protocol for network meta-analysis.

**Reply: I have registered according to your requirements, and the registration number has been added in the first chapter of the method.**

Additional Editor Comments:
I hope this message finds you well. Following a thorough review of the manuscript, it has come to our attention that there are several areas requiring revision and justification, as highlighted by the comments provided by the reviewers. To enhance the overall quality of your manuscript, we recommend seeking assistance from experts in Network Meta Analysis. Additionally, it would be beneficial to refer to the AMSTAR checklist to assess and ensure the robustness of your research.
Your attention to these aspects will significantly contribute to the refinement of the manuscript, aligning it more closely with the standards expected for publication.

**Reply: Thank you very much for your suggestion. I have made certain modifications according to the expert's requirements and also evaluated my research using the AMSTAR checklist.**

Reviewer #1: This paper underscores the importance of addressing insomnia in stroke patients and highlights the concerns surrounding the use of benzodiazepines for this purpose. Instead, it introduces eszopiclone (ESZ), a newer nonbenzodiazepine sedative, as a potential treatment option, given its favorable pharmacokinetics for stroke patients. The significance of this paper lies in its investigation of the safety and efficacy of ESZ when combined with Chinese medications for post-stroke insomnia treatment. It emphasizes the need for a systematic analysis of this condition, which has received limited attention compared to other stroke-related issues. Employing a network meta-analysis approach with outcome indicators like the Pittsburgh Sleep Quality Index (PSQI), this research can provide valuable insights for clinical practitioners, aiding them in informed decision-making for post-stroke insomnia treatment and potentially improving patient outcomes.

However, it's essential to acknowledge certain limitations of this study. Firstly, it exclusively focuses on randomized controlled trials (RCTs) involving ESZ-based drug combinations and excludes RCTs exploring alternative drug interventions due to the extensive existing literature. Secondly, many of the included studies lack essential details regarding concealment, blinding, and potential bias, which affects the overall reliability of the findings. Lastly, variations in treatment duration and baseline characteristics among the studies may introduce inconsistencies and clinical heterogeneity into the analysis, potentially impacting result validity. Nevertheless, this study marks a crucial step forward in understanding post-stroke insomnia treatment strategies.

However, there are several significant issues that need to be addressed for the manuscript to progress.

**Major Issues:**
1. It is essential to provide an explanation for the choice of keywords, particularly the omission of "nonbenzodiazepine" and similar mesh terms. The current set of keywords does not appear to result in a specific and comprehensive literature search.

**Reply: Your suggestion has been very helpful in adjusting my search strategy. I have revised the entire search strategy according to your requirements, as detailed in Appendix S1.**

2. Additionally, please include reasons for excluding specific types of original studies, such as observational cohorts, in order to justify the choices made.

**Reply: Thank you for your advise，The meta-analysis of comprehensive high-quality randomized controlled trials has been regarded as the highest level of evidence in evidence-based medicine. Therefore, this study only includes RCTs and excludes all other types of studies, as there are not too few included studies.I also added the reason to the exclusion section of the study.**

3. The manuscript should offer clear reasoning for opting for a fixed model when there was heterogeneity among studies, especially regarding specific treatments. Justification for this choice is crucial to ensure the robustness of the analysis.

**Reply: Thank you very much for correcting my mistake in choosing the model. I deeply understand that choosing a fixed model when there is heterogeneity is not appropriate, and have adjusted it to a random effects model.**

**Minor Changes:**
1. It is advisable to include a conclusion.

**Reply: The conclusion has been supplemented and modified according to your requirements.**

2. Figures 3, 4B, and 6 are currently of low resolution and readability.

**Reply: I have made corresponding modifications to the image quality in the article according to your requirements.**

3. Table captions should undergo revision to correct spelling errors. Additionally, in Table 1, please adjust the formatting for the entry "Song YM [19]" to ensure consistency.

**Reply: Spelling errors have been corrected and formatting has been adjusted.**

4. The keywords should be revised to eliminate repetitions and enhance clarity.

**Reply: I have made corresponding modifications to some keywords according to your requirements.**

Reviewer #2: I appreciate the authors' efforts in conducting this study; however, there are several critical concerns that need to be addressed before the manuscript can be considered for publication. Below are my detailed comments:

Abstract:

The use of uncommon and undefined abbreviations should be avoided in the abstract. The non-standard abbreviations used in the results section reduce readability and conciseness.

**Reply: I have defined the uncommon abbreviations in the abstract based on your suggestion.**

Introduction:
2. In line 6, the statement "there is still limited systematic analysis on poststroke insomnia" should be more specific. It would be more informative to highlight the shortcomings of previous meta-analyses on post-stroke insomnia rather than simply stating it as "limited."

**Reply: Your suggestion is very useful for my research, and I have made corresponding additions and modifications to this sentence based on your suggestion.**

Methods:
(1) The phrase "free words" is not commonly used; "keywords" is a more appropriate term.

**Reply: I have made corresponding modifications based on your suggestion.**

(2) It is unclear how the search terms were adjusted based on the search results. Was there a standardized process, or was it done subjectively?

**Reply: The search term is RCT, stroke, Insomnia, and Eszopiclone as the theme words, and their keywords are searched in the search library. Synonyms are connected by or, and different words are connected by and. Please refer to the appendix of S1 for specific search libraries.**

(3) The statement "If it was not calculated in the original text, it was calculated on its own" needs clarification. What does "calculated on its own" mean?

**Reply: I have added the self calculated formula to the manuscript.**

(4) The statistical methodology lacks detail and reproducibility. Please specify whether a frequentist or Bayesian network meta-analysis approach was employed, and provide information on the software packages or commands used.

**Reply: I have supplemented this section based on your suggestion and uploaded the software commands as an S2 appendix.**

Results:
(1) In the "Literature search results" section, it is mentioned that traditional Chinese medicine such as "shugan jieyu capsule (ESZ+SGJYC)" was used, but only the Pinyin transliteration is provided. This may not be clear to many readers and should be explained.

**Reply: Thank you very much for your insightful suggestion. We have provided a simple explanation for it.**

(2) Table 1 is missing essential patient characteristics such as disease duration and nationality.

**Reply: Thank you very much for your suggestion. Due to the limited space of the table, I have uploaded it as S1 table format.**

(3) The quality of figures is subpar with blurry text, poor screenshot quality, and messy formatting. It does not meet academic standards.

**Reply: I have made corresponding modifications to the image quality in the article according to your requirements.**

(4) The results section lacks specific numerical values and significance comparisons between therapies. The descriptions are mainly qualitative, and the numbers in the figures are barely readable. The paper needs to present numerical data for meaningful interpretation. Additionally, SUCRA values should be reported as numerical values, not just curves. The authors' network meta-analysis appears superficial, and they seem to lack familiarity with the standards for figure creation and academic writing in this field.

**Reply: The conclusion has been supplemented and modified according to your requirements.**

Conclusion:
5. The conclusion is too shallow and does not reflect the true clinical value and significance of the meta-analysis. It does not serve as a guidance for evidence-based medicine.

**Reply: The conclusion has been supplemented and modified according to your requirements.**

Other:

The English language used in the manuscript requires improvement. Expressions like "combined drug intervention therapy" and "clinical workers" sound unusual.
There are minor spelling errors, such as "Literature inclusion criteria" in the fourth line, which should be corrected.

**Reply: Thank you very much for your suggestion. I have made corresponding corrections to the English language and spelling issues in the manuscript.**
