# Supplementary material for: Efficacy and safety of eszopiclone combined with drug therapy in the treatment of insomnia after stroke: A network meta-analysis and systematic review
Source: PLoS One. 2024 Feb 5;19(2):e0297064. doi: 10.1371/journal.pone.0297064 (PMC10843102; doi:10.1371/journal.pone.0297064)
Supplement: Attachment — Submitted filename: rebuttal letter.docx [file pone.0297064.s007.docx]

Dear editor and reviewers

Journal Requirements:

Please review your reference list to ensure that it is complete and correct. If you have cited papers that have been retracted, please include the rationale for doing so in the manuscript text, or remove these references and replace them with relevant current references. Any changes to the reference list should be mentioned in the rebuttal letter that accompanies your revised manuscript. If you need to cite a retracted article, indicate the article’s retracted status in the References list and also include a citation and full reference for the retraction notice.

**Reply: I have checked all my references and ensured that they have not been withdrawn.**

Reviewer #4: Comments to the Submitting Author,

The authors evaluated the efficacy and safety of multi-drug therapy based on eszopiclone in the treatment of insomnia after stroke based on a network meta-analysis method. The results of this NMA provide some ideas for clinicians in practical applications and also provide some evidence for relevant decision makers in the development of guidelines. Currently, based on the previous peer reviewer's comments, the modifications made by the authors are acceptable. However, before formal publication, there are still some minor issues regarding citation of literature and resolution of images.

1. I am not sure if the authors of the 'Data extraction' chapter used the Cochrane Handbook as the source for their formulas, but it seems appropriate to cite the source of the relevant formulas.The formula is currently described in the latest version of the Handbook (https://training.cochrane.org/handbook/current/chapter-06#section-6-5-2-8):Plz cite this chapter as: Higgins JPT, Li T, Deeks JJ (editors). Chapter 6: Choosing effect measures and computing estimates of effect. In: Higgins JPT, Thomas J, Chandler J, Cumpston M, Li T, Page MJ, Welch VA (editors). Cochrane Handbook for Systematic Reviews of Interventions version 6.4 (updated August 2023). Cochrane, 2023. Available from [www.training.cochrane.org/handbook.](http://www.training.cochrane.org/handbook.)

**Reply: Thank you very much for your suggestion. I have already cited the references for the formula.**

2. At present, the RoB tool used by the author has certain problems, such as not considering special types of RCTs such as cluster randomization and crossover design, not considering the balance of inter group baselines, not clearly defining the effects of intervention allocation and compliance, and missing inter group contamination. Therefore, Cochrane launched a new version of the RCT Risk of Bias Assessment Tool (RoB2) in 2016, which became more comprehensive on the basis of RoB1. After multiple revisions, RoB2 has now become a widely applicable risk of bias assessment tool (https://training.cochrane.org/handbook/current/chapter-08#section-8-1). I hope that researchers can refer to the research methods introduced in the latest version of Cochrane Handbook to implement their own research in future related studies. In addition, please supplement the references of Cochrane Handbook in the 'Risk assessment' section.

**Reply: Your suggestions have been of great help to me, and I will refer to the research methods introduced in the latest version of the Cochrane Handbook in all my future research and i have supplemented the references of Cochrane Handbook in the 'Risk assessment' section**

3. Regarding the network plot in this article, the styles of the images uploaded by the authors seem to be different. Fig 5 has a background and background color compared to the other two.

In addition, can the authors optimize the layout of the images? Fig 3B and Fig 4B look very strange.

Finally, it is recommended that the author does not adjust the ratio of the length and width of the image, as both Fig 3 and Fig 4 seem to have this issue.
**Reply: I have made adjustments to the background and layout of Figure 3-5 based on your feedback.**
